# Appraisal of China's Response to the Outbreak of COVID-19 in Comparison With SARS

**DOI:** 10.3389/fpubh.2021.679540

**Published:** 2021-07-07

**Authors:** Jiajia Li, Shixue Li, Wuchun Cao, Zhongli Wang, Zhuohui Liang, Wenhao Fu, Jinfeng Zhao

**Affiliations:** ^1^Centre for Health Management and Policy Research, School of Public Health, Cheeloo College of Medicine, Shandong University, Jinan, China; ^2^National Health Commission Key Lab of Health Economics and Policy Research (Shandong University), Jinan, China; ^3^State Key Laboratory of Pathogen and Biosecurity, Beijing Institute of Microbiology and Epidemiology, Beijing, China; ^4^School of Public Health, Columbia University, New York, NY, United States

**Keywords:** infectious disease control, COVID-19, SARS, control measures, outbreak

## Abstract

Coronavirus disease 2019 (COVID-19), caused by severe acute respiratory syndrome coronavirus 2, was first reported in Wuhan, China, in December 2019 and has since become a pandemic. The COVID-19 containment measures were comparable to those used with severe acute respiratory syndrome (SARS), although these were stricter and more organized, and were initiated earlier and on a larger scale. Based on the lessons learned from SARS, the Chinese government acted aggressively in response to COVID-19, through a unified and effective commanding system, using law-based and science-driven strategies, and coordinated deployment of medical resources. Additionally, the application of high-tech measures, traditional Chinese medicine, and hierarchical medical systems also played an important role in control measures. Despite the remarkable performance, the initial delay in response suggests that the coordination between public health and medical services, reserve and coordination of emergency materials, and capacity for disease control and prevention need to be strengthened.

## Introduction

In December 2019, a novel coronavirus, severe acute respiratory syndrome coronavirus 2 (SARS-CoV-2), which causes coronavirus disease 2019 (COVID-19), emerged in Wuhan, China. By December 21, 2020, the number of COVID-19 cases worldwide reached 75,704,857 with 1,690,061 deaths.

As the first country affected by COVID-19, local outbreaks have been largely contained in mainland China. The prompt and decisive response to COVID-19 in China was hailed as an extraordinary achievement by the World Health Organization (WHO) and “bought time” for the development of an effective vaccine and implementation of other measures in response to COVID-19 (1). Dr. Tedros praised China for its transparency, data sharing, and quick response (2).

Adopting a similar approach as the response to SARS, COVID-19 was mainly contained through traditional public health interventions, such as case detection and isolation, close contact tracing and quarantining, social distancing, screening of travelers, implementation of infection prevention guidelines, and enhanced infection control in healthcare settings. In contrast to SARS, the total number of COVID-19 cases is much higher due to the inherent difficulties in identifying and counting mild and asymptomatic cases (3). The objective of this review is to explore how China has responded to the challenges presented by COVID-19 and what new lessons have been learned from the COVID-19 response.

## Method

We based our review on reports (international and domestic), official documents, and published work. We searched the Web of Science, PubMed, and China Knowledge Resource Integrated Database for articles, books, and reports published from 2003 onwards. In addition, official websites of the Chinese government, National Health commission of China, and Chinese Centre for Disease Control and Prevention (CDC) were searched for information, official documents, and guidelines. The Report of the WHO-China Joint Mission on Coronavirus Disease 2019 (COVID-19) and other information issued by the WHO served as additional sources. We restricted our search to works published in English or Chinese and used the following search terms: “control measures,” “containment measures,” “public health measures,” “response,” “SARS,” “COVID-19 or 2019-nCOV,” “China,” and combinations of these terms. The date of the last search was February 25, 2021. Moreover, a book published by the Chinese CDC in 2003, “Compilation of literature on prevention and control technology and integrated management of SARS,” provided evidence regarding important moments during the SARS epidemic.

## Results

**Figure 1:**
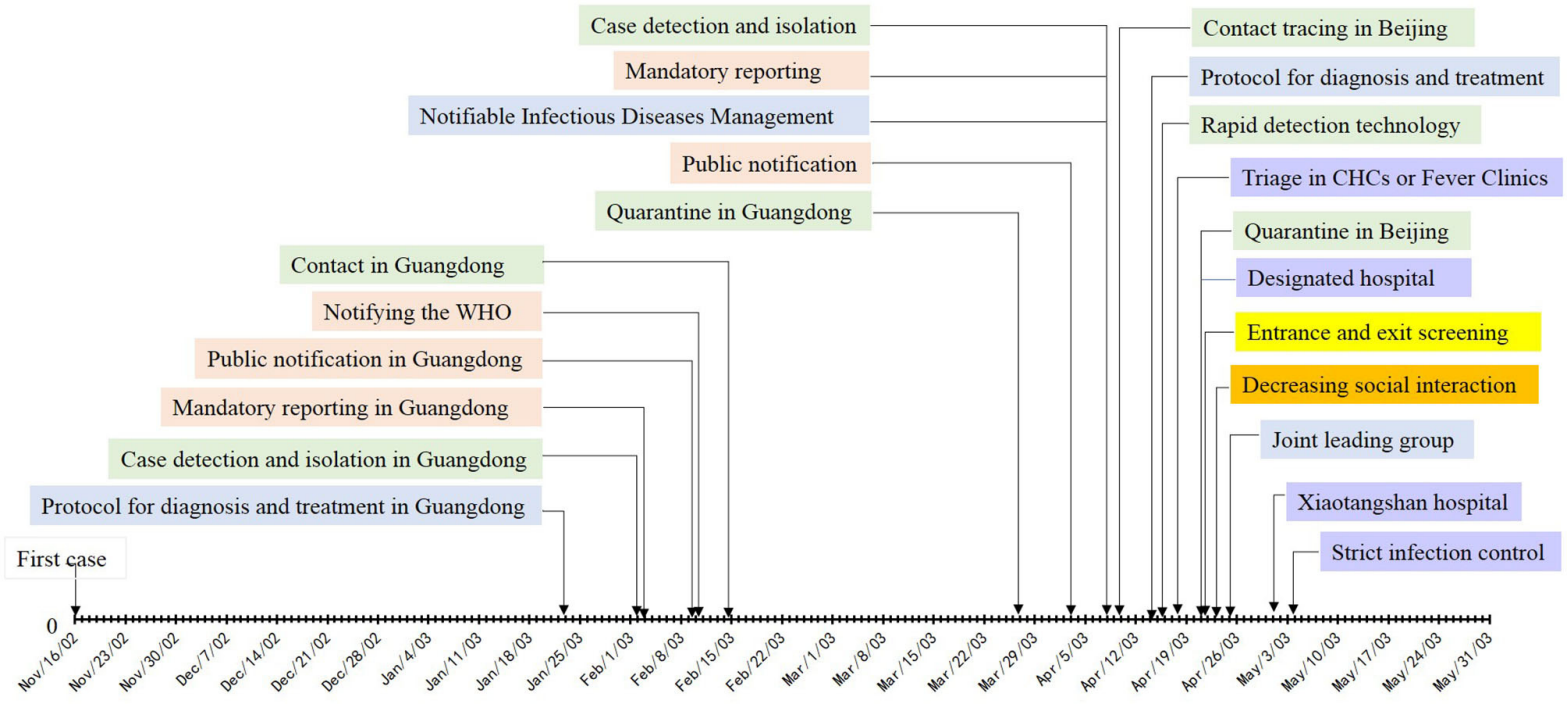
Timeline of control measures implemented in SARS.

**Figure 2:**
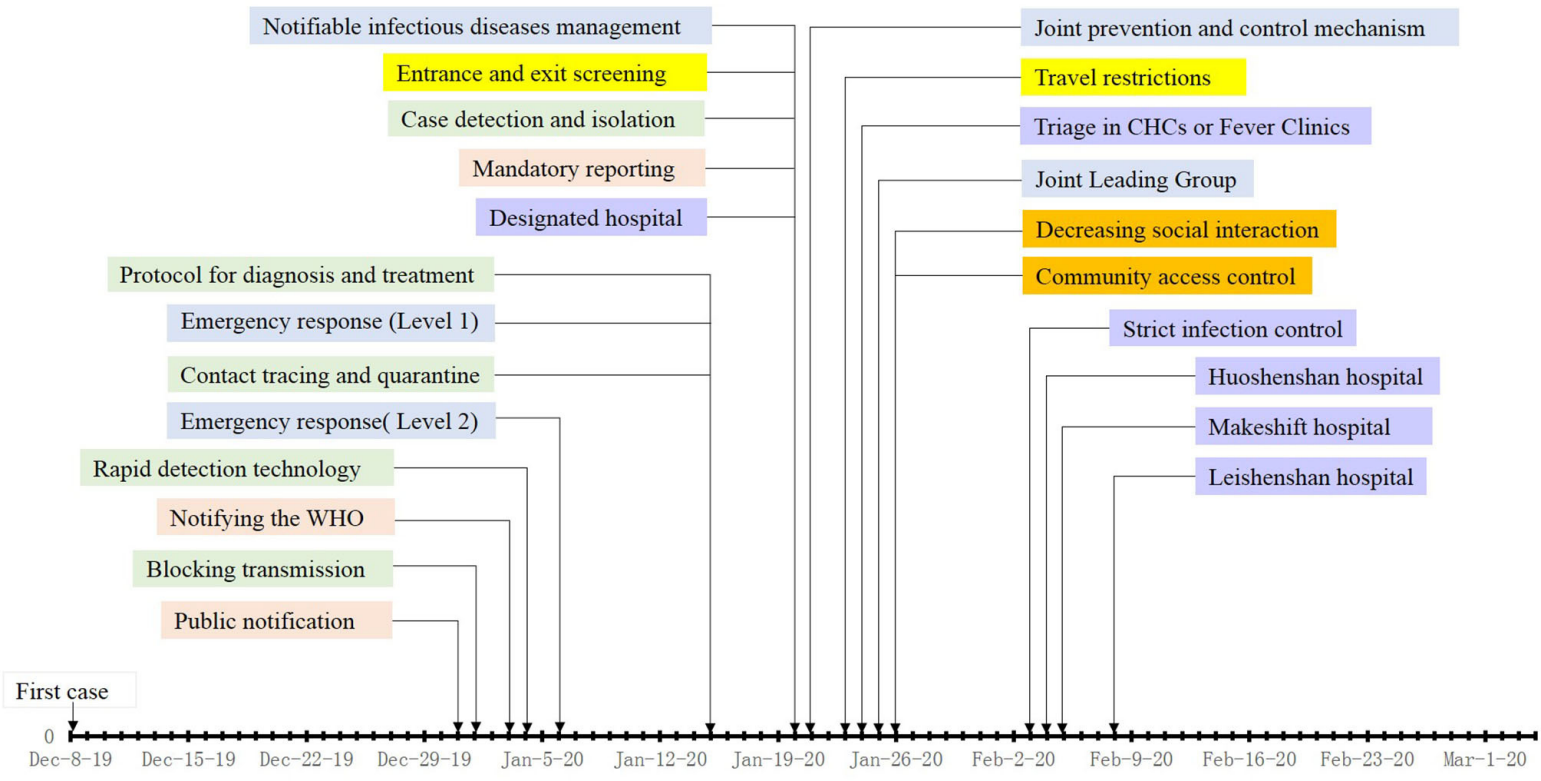
Timeline of control measures implemented in COVID-19.

Table 1 summarize the timeline of outbreaks and control measures implemented during the outbreaks of SARS and COVID-19 in mainland China. This shows that although similar containment measures were applied during the two outbreaks, these were initiated at a later stage and on a smaller scale during the SARS outbreak. Figures 1, 2 more intuitively show the difference in response speed between COVID-19 and SARS. During the SARS outbreak, state-level control measures were not implemented until February 2003. It was only after a second outbreak in Beijing in April 2003 that a nationwide response began. In stark contrast, the Chinese government initiated large-scale and forceful control measures within 2 months of the COVID-19 epidemic. The following six control categories were analyzed to explore what had changed since the 2003 SARS outbreak, and how exactly have these changes had an effect with respect to the response to COVID-19, regardless of whether these changes were an improvement or setback.

**Table 1:** Summary of the main control measures implemented during SARS and COVID-19, by date of onset (if available).

	**COVID-19**	**SARS**
**Date of onset**	8 December 2019	16 November 2002
**Number of cases reported**	90.655 (Mainland, by 29 April 2021)^a^	5.327 in mainland China
**Control measures**	**Days since onset (Date)**	**Days since onset (Date)**
Organizational and administrative measures		
Joint prevention and control mechanism	44 days (21 January 2020)	N/A
Joint leading group	48 days (25 January 2020)	158 days (25 April 2003)
Emergency response	Level 2: 29 days (6 January 2020) Level 1: 38 days (15 January 2020)	N/A
Notifiable infectious disease management	43 days (January 20, 2020)	143 days (8 April 2003)
Reporting		
Public notification	23 days (31 December 2019)	138 days (3 April 2003); Guangdong-86 days (10 February 2003)
Mandatory reporting	43 days (20 January 2020)	143 days (8 April 2003); Guangdong−79 days (3 February 2003)
Notifying the WHO	26 days (3 January 2020)	87 days (11 February 2003)
Case detection and contact tracing		
Blocking transmission	24 days (1 January 2020)	N/A
Protocol for diagnosis and treatment	38 days (15 January 2020)	149 days (14 April 2003); Guangdong-68 days (23 January 2003)
Rapid detection technology	27 days (4 January 2020)	151 days (16 April 2003)
Case detection and isolation	43 days (20 January 2020)	143 days (8 April 2003); Guangdong 77 days (1–3 February 2003)
Contact tracing and quarantine	38 days (15 January 2020)	
Contact tracing		Guangdong- 77 days (early February 2003); Beijing-144 days (9 April 2003)
Quarantine		Guangdong- 131 days (27 March 2003); Beijing 156 days (21 April 2003)
Travel-related measures		
Travel restrictions	46 days (23 January 2020)	N/A
Entrance and exit screening	43 days (20 January 2020)	157 days (22 April 2003)
Community containment measures		
Decreasing social interaction	49 days (26 January 2020)	158 days (23 April 2003)
Community access control	49 days (26 January 2020)	Only in very few communities
Hospital containment measures		
Strict infection control	57 days (3 February 2020)	169 days (4 May 2003)
Establishing separate triage facilities		
Triage in CHCs or Fever Clinics	47days (24 January 2020)	152 days (17 April 2003)
Designated hospital	43 days (20 January 2020)	156 days (21 April 2003)^b^
New hospital	Huoshenshan 58 days (4 February 2020) Leishenshan 62 days (8 February 2020)	Xiaotangshan 166 days (1 May 2003)
Makeshift hospitals	59 days (5 February 2020)	N/A

a *Data from the website of National Health commission of China: http://www.nhc.gov.cn/xcs/yqtb/202104/80fb5915f82049f4abf53293804382a2.shtml. (accessed April 30, 2021)*.

b *http://news.sina.com.cn/c/2003-04-21/20201008906.shtml*.

### Organizational and Administrative Measures

#### Joint Prevention and Control Mechanism

A unified and effective command system is a prerequisite for the effective and orderly implementation of prevention and control measures (4). In response to the SARS and COVID-19 outbreaks, the Chinese central government set up task forces to coordinate national infection control efforts, although response timelines varied widely. The national “SARS control and prevention headquarters” was set up only when the outbreak affected Beijing (5), by which time the infection had spread to 26 provinces in China (6). The lack of coordination in the early stages of the SARS outbreak led to extensive spread. Based on the earlier experience of SARS outbreak, as soon as human-to-human transmission was identified, the State Council established a Joint Prevention and Control Mechanism (JPCM) composed of 32 ministries (7). A central leading group on epidemic response and a central steering group stationed in Wuhan were established on January 25, 2020, with Prime Minister Li Keqiang as the leader and Vice-Premier Sun Chunlan as the frontline leader. Furthermore, President Xi Jinping personally presided over China's epidemic response to COVID-19, which greatly increased the commitment of and leadership from the government to respond to public health emergencies (1).

#### Emergency Response

During the SARS outbreak, China had no legal mechanism to directly oversee public health emergencies (8). On May 12, 2003, the State Council promulgated the “Regulations on Public Health Emergencies,” which stipulates the responsibilities that governments at all levels and medical institutions should assume at various stages of public health emergencies (9). In 2006, the government issued the “National General Guideline on Public Health Emergencies” to standardize the process of dealing with public health emergencies, which divided the public health emergencies into four levels (10). In accordance with the above laws, the Chinese CDC initiated Level 2 and Level 1 emergency responses on January 6 and January 15, respectively (11). As of January 25, 2020, 30 provinces in mainland China initiated a Level 1 emergency response (7), meaning the Stata Council has unified command of emergency response.

#### Notifiable Infectious Disease Management

Both SARS and COVID-19 were added to the list of notifiable infectious diseases. While SARS took nearly 5 months to be considered a statutory infectious disease, COVID-19 was defined as a Class B notifiable disease in 43 days (12); Subsequently, it was managed as a Class A infectious disease. This means that SARS and COVID-19 were required to be managed using the prevention and control measures of Class A infectious diseases, such as mandatory reporting and isolation of confirmed and suspected cases (13). COVID-19 was also defined as a quarantinable communicable disease to control international spread (14).

### Reporting

#### Public Notification

On February 10, 2003, the Guangdong Ministry of Health (MOH) held a press conference to announce an outbreak of atypical pneumonia (6, 15). This took place 87 days after the first identified case of SARS and, at this point, 218 cases had been detected in Guangdong (6). Prior to this and for the month following the announcement, the SARS epidemic went largely unnoticed, likely resulting in many avoidable cases (16)^1^. Based on earlier experience and more recent advances in the public health system, on December 31, 2019, 27 cases of pneumonia of unknown etiology were publicly reported by the Wuhan NHC for the first time (3). Notification of the COVID-19 outbreak to the public, which occurred ~23 days from the COVID-19 onset, occurred more rapidly than in the earlier SARS outbreak. Official daily disease information was released on January 21, 2020. The JPCM held a daily press conference to share the latest news with the public from January 27 (7).

#### Mandatory Reporting

In China's Guangdong Province, reporting of cases of atypical pneumonia using a standard case definition and reporting form became mandatory from February 3, 2003 (17). At the beginning of the spread of SARS, due to the lack of transparent reporting system, information regarding the outbreak was not clear; it took 8–9 days for a detected case to be reported and 3–4 days for a hospitalized case to be reported, seriously affecting the ability to implement timely control measures (18). After SARS was included in the statutory report of infectious diseases on April 8, 2003, health authorities in all provinces were required to collect and report all probable cases and deaths using a standardized case report form (19). To avoid previous errors, the Reporting Regulations for Public Health Emergencies and Communicable Diseases Surveillance was issued in November 2003, clearly set out the required information and time limits for the reporting of infectious disease outbreaks and epidemics (20). Mandatory reporting of COVID-19 has been in place since the introduction of notifiable infectious disease management in January 20, 2020 (21). This involves reporting of all suspected cases, confirmed cases, and asymptomatic infected individuals *via* a web-based reporting system within 2 h of diagnosis. Violating reporting rules or concealing information is punishable according to law (22).

#### Notifying the WHO

The MOH and CDC notified the WHO about the SARS outbreak on February 11, 2003, 3 months after the first case was reported, when 300 cases and five deaths had already occurred (3). One month later in March 12, 2003, when the number of cases reached ~800 in mainland China (23), the WHO issued global alerts (4, 23). The WHO was notified of COVID-19 on January 3, 2020 (24). At this point, there were only 27 known cases and zero deaths (3). The WHO shared the detailed information and issued a Disease Outbreak News report on January 5, and then declared COVID-19 a Public Health Emergency of International Concern on January 30, 2020. The WHO expressed its satisfaction with the swift and effective information sharing by China (25) as this greatly helped in coordinating international resources to address emerging problems.

### Case Detection and Contact Tracing

#### Controlling Sources of Infection

On January 1, 2020, the government shut down the probable infection source, Huanan Seafood Wholesale Market, as links between infected individuals and the market were found in most of the 41 laboratory-confirmed cases (3, 26). The 4Es, consisting of early detection, early reporting, early isolation, and early treatment, were subsequently implemented nationwide and greatly contributed to controlling the spread of disease (7). In contrast, the palm civet was not removed from the markets until it was identified as a reservoir in the SARS outbreak (26).

#### Case Detection and Isolation

Case detection and isolation is an effective measure to limit community transmission of infectious disease. By April 16, 5 months after the first case was identified, rapid detection technology had been developed that could provide results within 2 h of testing. Due to the lack of accurate guidelines and detection technology in the early stage of the SARS epidemic, 709 cases occurred in healthcare workers (HCWs) (19). Following significant progress, on 4 January 2020, 4 days after isolating the virus specimen, the Chinese CDC successfully developed polymerase chain reaction diagnostic reagents that were used for the detection and diagnosis of suspected COVID-19 cases in Wuhan on January 11 (11). To avoid missed detection, the NHC updated the diagnostic criteria to include suspected cases in Hubei with imaging features of pneumonia (27).

For early case detection, it is necessary to provide accurate case definition and diagnostic protocols for hospitals, in addition to rapid detection technology. On April 3, 2003, almost 5 months after the first SARS case was detected, the Chinese CDC officially released the diagnostic criteria and treatment protocols for SARS on a nationwide scale (19). Actually, Guangdong authorities developed and implemented treatment and control guidelines as early as January 2003, and these were praised by the WHO as a “model for the rest of China or maybe for the rest of the world;” (4) however, these guidelines were not promptly or fully shared with other provinces in China or other countries (28). Taking into account the inadequate response to SARS, the National Health Commission (NHC) issued the first edition of the Diagnosis and Treatment Protocol for COVID-19 on January 15, 2020, only 12 days after the WHO had been notified about the new virus. By March 4, the Protocol was updated to the seventh edition (Table 2), as knowledge of the virus and experience in the diagnosis and treatment of COVID-19 accumulated.

**Table 2:** Comparisons of seven editions of Diagnosis and treatment Protocol for COVID-19.

**Edition**	**Date**	**Revisions**
Edition 1–2	January 15–16	–
Edition 3	January 22	Epidemiology- History of travel to or residence in Wuhan and its surrounding areas, or in other communities where cases have been reported within 14 days prior to the onset of the Disease; or in contact with novel coronavirus infected people (with positive results for the nucleic acid test) within 14 days prior to the onset of the disease. Clinical performance- Fever, fatigue and dry cough were the main performance. Some patients may not have fever Diagnostic criteria- The COVID-19 is highly homologous with the known gene sequence if the nucleic acid positive Refined treatment plan.
Edition 4	January 27	Epidemiology-Add clinical classification
Edition 5	February 4	Epidemiology-The infection source was COVID-19 patients and asymptomatic infectious. Clinical performance-Added severe and mild patient symptoms. Diagnostic criteria-Different treatment between Hubei Province and other provinces except Hubei Province 1. Treatment-Added “no effective antiviral therapy has been confirmed at present” and “people with condition can conduct cytokines detection”
Edition 6	February 18	Epidemiology-Added the possibility of aerosol transmission. Diagnostic criteria- canceled the difference between Hubei Province and other provinces, which can be divided into “suspected cases” and “confirmed cases.” Treatment-Added trail drugs and divide TCM plan
Edition 7	March 3	Epidemiology- Novel coronavirus can be isolated in feces and urine, attention should be paid to feces or urine contaminated environment that may lead to aerosol or contact. Clinical performance- Added the description of clinical manifestations of pregnant women and children and serological test. Diagnostic criteria- Make explanation of “clustering disease” in epidemiology and modify the content “Lymphocyte count decreased” in clinical manifestation s to “lymphocyte count was normal or decreased.”

During the SARS outbreak, cases in Guangdong were required to isolate from the beginning of February 2003 (17). However, in Beijing, isolation of cases was not commenced until SARS was included as a statutory infectious disease on April 8 (19). However, Wuhan isolated confirmed and suspected COVID-19 cases from January 20, 2020, according to the Law of the People's Republic of China on prevention and control of infectious diseases (2).

#### Contact Tracing and Quarantine

During the SARS outbreak, contact tracing and quarantine activities were mainly initiated in the epidemic areas, Guangdong in early February, and Beijing in early April (17). Beijing quarantined close contacts individually and in groups from 21 April, 2003 (19), among which 12,000 people were quarantined in completely sealing off hospitals, construction sites, residential buildings, and universities (29). COVID-19 is contagious during its incubation period and, accordingly, a stricter and more extensive quarantine of contacts was implemented. Since the first edition of the Prevention and Control Protocol for COVID-19 was issued on January 15, 2020, all close contacts were under medical observation at home or at designated places (e.g., hotels), with no permission to undertake unnecessary outdoor activities. Many provinces quarantined all returnees from Hubei province for 14 days, even if they had no contact with any confirmed cases (30). New technologies such as big data and artificial intelligence (AI) have been applied to strengthen contact tracing and the management of priority populations (1). For example, the fellow traveler inquiry system was used to check whether passengers had a history of traveling with any confirmed cases so that close contacts could be identified or self-reported. With the increase in cluster outbreaks, many provinces no longer quarantined close contacts at home but placed them in dedicated sites for medical observation (31).

### Travel-Related Measures

#### Travel Restrictions

Due to the travel season peaking during the Spring Festival holiday in China, travel restrictions during the COVID-19 outbreak were more stringent and extensive than those for SARS. On January 23, all transportation networks to and from Wuhan were shut down, including railway stations, airports, and bus stations. Subway and bus services in Wuhan were also suspended (32). By January 25, restrictions were expanded to other cities in Hubei Province, prevented the virus from spreading outside Hubei. Although there were stricter requirements for travel to and from the affected areas during SARS (22), there were no control as stringent as city-level lockdowns.

#### Entrance and Exit Screening

In the early stages of the SARS epidemic, there were no clear travel advice or precautions. On April 12, 2003, 147 days after the onset of the SARS outbreak, the former Ministry of Health (MOH) and five other departments jointly issued a notice to begin travel-related measures to prevent and the spread of SARS by means of transport (33). From April 22, 2003, all arriving and departing passengers were required to submit health declaration cards and undergo temperature checks. During the COVID-19 outbreak, through continuous improvement of the legal system, travel-related measures, such as temperature checks and healthcare declarations, were implemented at transportation hubs on January 20, 2020, by which time few cases had been confirmed outside Wuhan (34). Meanwhile, the JPCM published guidelines recommending the use of masks on public transport and providing advice on the disinfection of transportation depots (35).

### Community Containment Measures

#### Decreasing Social Interaction

Measures aimed at increasing social distance were implemented in epidemic areas during the SARS outbreak; these were applied nationwide during the COVID-19 epidemic. From April 24, 2003, schools and public places were closed in Beijing (19). Meanwhile, 22 (32%) of the 68 universities in Beijing canceled classes and allowed limited visits (19). The COVID-19 outbreak coincided with the Spring Festival holiday, the most popular time for Chinese family gatherings and public entertainment. To avoid cluster transmission, the Spring Festival holiday has been extended to 10 days on January 26, 2020 (36). Subsequently, the Ministry of Education issued a postponement notice of the new academic semester on January 27 (37). Meanwhile, the JPCM advised people to stay at home; canceled large mass gatherings, such as lantern shows during the Lantern Festival; and closed public places, such as libraries, cinemas, shopping malls, and parks.

#### Community Access Control

During the SARS outbreak, closed-off community management was mainly implemented in areas where extensive unexplained community transmission was suspected (38). During COVID-19, community containment was implemented as soon as the first cases of COVID-19 were found outside of Wuhan on January 15 (7). From January 26, 2020, the day after the Spring Festival, strict access control to all communities and villages was initiated. With the community as the basic unit, China conducted nationwide grid management to ensure 4 earlies (7). The population was urged to check and report their temperature to their community grid staff and employers at least once a day. Thermal scanning at the point of entrance to the community was implemented in almost every community, not just in those where community transmission was suspected.

### Hospital Containment Measures

#### Strict Infection Control

On May 4, 2003, the MOH developed detailed SARS infection control guidelines for both hospitals and HCWs and conducted special infection control training courses for HCWs. Beginning on April 18, prior to the MOH's announcement, 62,363 health care workers in Beijing underwent training on the management of patients with SARS, infection control, and the use of PPE through in-person courses, videotapes, and printed materials (19). However, the training activities seemed to have been supplied too late. By May 2, 2003, 778 SARS cases were identified as nosocomial infections, accounting for over 20% of the total 3,799 cases (19). In light of the previous experience with SARS, the NHC issued guidelines for infection control and prevention techniques and the use of PPE in medical facilities in the early stages of the COVID-19 outbreak (39). Furthermore, medical institutions across the country enforced an emergency pre-examination triage system on February 3, 2020. Many hospitals launched online consultations, set up emergency isolation areas in general wards, and tightened visitation to prevent the infection of other patients (31).

#### Establishing Separate Triage Facilities

A hierarchical treatment system was established for both SARS and COVID outbreaks, comprising fever clinics, designated hospitals, and new specialized hospitals for patient triage, isolation, and treatment. On April 17, 2003, 123 fever clinics were established in all secondary and tertiary hospitals in Beijing (19). On April 27, all patients with SARS were placed together in designated hospital wards (19). A new 1000-bed SARS hospital, Xiaotangshan Hospital, was opened on May 1 (40).

Unlike SARS, primary medical institutions played an important role in the triage of COVID-19. Of the 203 Community Health Centers in Wuhan, 199 were designated for COVID-19 screening and triage on January 24 (41). After the SARS outbreak, the former MOH required all hospitals above level II to regularly set up infectious diseases departments that included fever clinics, which were separate from other patient care areas and staffed by trained personnel (42). As the epicenter of infection, Wuhan released a list of the first group of designated hospitals with fever clinics on January 20 (41). Huoshenshan Hospital and Leishenshan Hospital, which are mainly used for treating severe patients, started to treat patients on February 4 and February 8, respectively (41). In addition, public places were transformed into makeshift hospitals starting on February 5 to isolate and treat many patients with mild disease. In total, 16 makeshift hospitals treated over 12,000 patients in Wuhan (43).

## Discussion

Evidence from previous studies indicates that strong political commitment and a centrally coordinated response were the most important factors underlying the control of SARS in mainland China (44). Although there were effective containment guidelines at the time of SARS, the government's hesitation resulted in a nationwide outbreak of SARS in China. Following the disastrous experience of SARS, the Chinese government acted aggressively during the COVID-19 outbreak, implementing decisive measures, such as a cordon sanitaire around Wuhan, restriction of mass gatherings, and prolonged holidays. Previous studies have shown that the drastic control measures implemented in China substantially mitigated the spread of COVID-19 (45). These measures should be properly recognized as the situation may have been worse if these measures had not been implemented to respond to COVID-19 during a period of general population mobility in China. There were both improvements and new lessons in disease prevention and control.

### Achievements

#### Law-Based Strategies

After the SARS outbreak, the central government revised the law on the control of infectious diseases in March 2004.The revision provides instructions to respond to infectious disease outbreaks, improve the reporting of infectious diseases, implement interventions to control disease spread, provide clinical services, fund the control of infectious diseases (46). In 2007, the Emergency Response Law of the People's Republic of China was issued and further stipulated the establishment of an emergency management system that urged unified leadership, comprehensive coordination, categorized management, graded responsibility, and territorial management (47). As a result, the government's response to the COVID-19 outbreak was organized and transparent.

#### Implementing the National Reporting System (NRS)

On April 1, 2004, the MOH implemented the world's largest Internet-based communicable-disease reporting system, which was jointly funded by the central government (250 million CNY) and local governments (480 million CNY). This system addressed the delays and incomplete reporting of communicable diseases, which were most evident during the SARS epidemic when governmental authorities could not quickly assess the extent of the epidemic. Up to April 2014, all CDCs at different levels, 98% of health facilities at and above the county level, and 94% of township-level health facilities reported the country's 39 notifiable diseases through this system (48). The mean length of time to report from a county-level health facility to the central level was reduced from 29 days to 1 day (49). COVID-19 was integrated into the system on January 24, 2020 (50), and official figures were published daily, which provided governments and their respective departments with an up-to-date understanding of the situation, allowing evidence-based changes in their control measures (7). De facto, the daily report delivered correct and timely information to the public, which prevented mass panic and helped the public protect themselves (51).

#### Strengthening CDC Systems

The Chinese government has devoted substantial resources to developing a new CDC system after SARS (52). This was re-formed in 2006 into a four-level (i.e., central, provincial, city, and township) disease control and prevention as well as health surveillance system. There has been substantial investment in public health infrastructure, such as new buildings, improvements in internet connectivity, and the purchase of advanced equipment (16). By 2012, CDCs across China had received 93 billion and 8 million CNY, which increased by 516.8% during the decade. There was an annual expenditure of 2.7 million CNY for the prevention program, which increased by 821.4% over the same period (53). Significant improvements in the capacity of public health personnel have been achieved in the workforce-development program through the Field Epidemiology Training Program.

### Highlights

#### Extensive Use of Traditional Chinese Medicine

The combination of traditional Chinese medicine (TCM) and Western Medicine (WM) was extensively employed for the treatment of both SARS and COVID-19. At the time of SARS epidemic, 58.27% of the clinically confirmed patients with SARS received TCM treatment in China, with apparent curative effect (54). On March 6, 2020 at the State Council Press Conference, Yu Yanhong, a member of the Central Leadership Group, made the following statement: “The fact that most of the 50,000 cured patients have adopted TCM, fully proves that the integrative TCM/WM has a remarkable effect” (30). Based on the curative effects of TCM in patients with COVID-19, dozens of provinces have published COVID-19-related prevention and treatment guidance for TCM (31). TCM was used in over 99.93% of cases in the makeshift hospitals in Wuhan (41).

#### A Hierarchical Medical System Helped to Control the Outbreak

All suspected cases were first screened, classified, and located in the community. Highly suspected cases were then transferred to fever clinics for further medical examination, while quarantine and isolation at home was imposed for other cases. Patients with severe symptoms were transferred to specialized hospitals. This triage process reduced the risk of cross-infection and reduced the pressure on COVID-19-designated hospitals.

#### Application of High-Tech Measures

Many forms of eHealth services have been implemented during the COVID-19 outbreak, such as online outpatient services, online COVID-19-related consultations, and AI doctors. In addition to medical services, some services used health QR codes, such as health condition checks and community entrance passes, during the outbreak (51). After the initial outbreak between December 2019 and March 2020, smaller-scale resurgences occurred in Beijing and Heilongjiang, among other places (55). Targeted “test-trace-isolate” strategies was adopted during these resurgences. Big data and AI technology played a role in the determination of population mobility, the conduct of epidemiological research, and the tracing close contacts (1, 43, 51).

### Challenges

#### Initial Delay in Information-Sharing

In December 2019, some hospitals reported cases of unexplained pneumonia to the Wuhan Health Committee. However, it was not until January 14, 2020 that a surveillance system for COVID-19 was integrated with an internet-based infectious disease reporting system (50). However, some blame the convoluted process for reporting cases and the lack of practitioner training for the spread of misinformation in the early stages of the outbreak (25).

#### Disconnection Between Disease Prevention and Treatment

The CDC in China is classified as a public institution that has no authority with respect to public affairs, including public health emergencies. In addition to the lack of decision-making authority, the CDC in China failed to cooperate with the medical system. CDC professionals are only allowed to have licenses for public health practitioners, which forbids the issuing of prescriptions and conducting clinical work, while clinical practitioners are allowed to diagnose and prescribe; however, they lack experience in infectious disease testing, investigation, and reporting. This disconnect between public health and clinical practitioners from these two systems resulted in neglect and failure in containing the outbreak at an early stage.

#### Urgent Work Needed to Strengthen Disease Control and Prevention System

China was applauded for its progress in improving the disease prevention system since the SARS outbreak; however, in 2012 the follow-up reform that changed the CDC into a non-profit public institute resulted in cutbacks in both personnel number and income. Statistics show that the brain drain from different levels of CDC greatly increased between 2009 and 2017. The number of public health personnel dropped by 4.1% (56). Meanwhile, township CDC staff appear to be inadequately qualified, with only 10.7% of personnel having senior titles and only 25% of personnel holding a bachelor's degree or above (53). In addition to the lack of professional staff, there was a shortage of equipment. Only 20% of provincial CDCs were equipped with a minimum number of Standard-A hardware (56). Consequently, the CDC failed to fulfill its mission during this outbreak.

## Conclusions

As the first country to experience both the SARS and COVID-19 epidemics, attention was garnered by the public health system in China and interventions were taken to improve it. The SARS outbreak clearly highlighted weaknesses of the public health system and emergency management system. Therefore, once the outbreak ended, the government prioritized strengthening of the CDC systems, improving the legal system, and implementing an internet-based communicable-disease reporting system. Based on the lessons learned from tackling SARS, the COVID-19 containment measures were stricter and more organized, and were initiated earlier and on a larger scale than those used with SARS. Although China has made great progress, as can be seen in its response to COVID-19 in comparison with that to SARS, some exposed weaknesses suggest that further efforts should be made to improve the capacity of the disease prevention and control systems. First, the CDC's staffing, equipment and financial support should be ensured. Second, the CDC's integration with medical institutions regarding disease prevention and treatment should be strengthened. Third, the information sharing mechanism between regions and departments should be improved.

## Data Availability Statement

The original contributions presented in the study are included in the article/supplementary material, further inquiries can be directed to the corresponding author/s.

## Ethics Statement

This study was approved by Academic Research Ethics Committee of school of public health, Shandong University.

## Author Contributions

SL and WC are responsible for the overall design of the review and analysis of the paper. JL wrote the first draft of the manuscript and contributed to the production of the figures. ZW produced Table 1, contributed to the literature review, and analysis of the paper. WF and JZ contributed to the figures and literature review. WC edited the manuscript and contributed to the overall interpretation of the findings. ZL edited the manuscript and contributed to the literature review and analysis. All authors contributed to the literature review, data gathering and analyses, and comments on the manuscript.

## Conflict of Interest

The authors declare that the research was conducted in the absence of any commercial or financial relationships that could be construed as a potential conflict of interest.

## Footnotes

^1^Summary of SARS prevention and control work[EB/OL] http://www.gov.cn/test/2005-06/28/content_10716.htm.

## Abbreviations

COVID-19: coronavirus disease 2019
WHO: World Health Organization
SARS: severe acute respiratory syndrome
JPCM: Joint Prevention and Control Mechanism
MOH: Ministry of Health
NHC: National Health Commission
HCWs: health care workers
AI: artificial intelligence
TCM: traditional Chinese medicine
SHI: Social Health Insurance
NHSA: National Healthcare Security Administration.
